# Strengthening effectiveness evaluations through gender integration to improve programs for women, newborn, child, and adolescent health

**DOI:** 10.1080/16549716.2021.2006420

**Published:** 2022-09-13

**Authors:** Rosemary Morgan, Henri Garrison-Desany, Amy J. Hobbs, Emily Wilson

**Affiliations:** Department of International Health, Johns Hopkins Bloomberg School of Public Health, Baltimore, MD, USA

**Keywords:** Gender, gender integration, women’s health, maternal health, child health

## Abstract

Over the past decade, there has been substantial progress towards integrating our understanding of social determinants of reproductive, maternal, newborn, child, and adolescent health (RMNCAH) into health planning and programs. For these programs, gender inequity remains one of the most harmful factors for women’s access to healthcare. Designing RMNCAH programs to be gender-responsive through active engagement with and overcoming of gender-related barriers remains paramount to increasing women’s access to and use of health programs. However, the integration of gender within RMNCAH programs and their evaluation is often non-existent or is limited in scope. Building on a prior framework for comprehensive gender analysis in RMNCAH, we discuss key steps used to incorporate a gender lens and analytical approach in the Real Accountability: Data Analysis for Results (RADAR) evaluation framework. In order to effectively address these key areas, gender must be integrated into all stages of the evaluation, including tool development and programmatic activities that are associated with each question. Our paper includes practical activities and tools that evaluators may use to integrate gender into their evaluation tools.

## Background

Over the past decade, there has been substantial progress towards integrating our understanding of social determinants of reproductive, maternal, newborn, child, and adolescent health (RMNCAH) into health planning and programs. In addition to understanding social context in relation to maternal outcomes, including maternal health care delivery [1], and sexual and reproductive health outcomes, including HIV prevention and care [2], attention has also been paid to acknowledge and address the social inequalities that lead to health inequalities. Of particular note for RMNCAH programs, inequality on the basis of gender remains a major barrier that limits women’s access to healthcare.

For RMNCAH programs to be gender-responsive, they need to overcome gender-related barriers that reduce women’s access to and utilization of health programs [3,4]. In order to integrate gender into program activities, one must use a broad definition of gender, and consider the gendered power dynamics that are at play in the target population [5]. Here gender is defined as the ‘roles, behaviors, activities, attributes, and opportunities that any society considers appropriate for girls and boys, and women and men’ [5,6]. Additionally, individuals may identify with neither or both of these gender categories throughout their lifespan.

Prior studies and frameworks have highlighted that gender power relations are multi-level, impacting not only individuals, but manifesting throughout different levels within social networks, including the family, wider community, and the healthcare system [5,7]. Therefore, it is important that RMNCAH programs consider and incorporate a gender lens from the initial planning stages through to the final program and within the program’s monitoring and evaluation framework.

However, the integration of gender power relations within RMNCAH programs and their evaluation is often limited in scope. Rather than assessing gender dynamics using a multi-level framework, many programs only present outcome-level estimates disaggregated by sex. Even among gender-specific interventions, such as those increasing male partner involvement in maternal and child health, gender is often discussed with a single individual at a time, rather than a holistic understanding of gender in the context of a relationship/marriage. Additionally, these analyses rarely examine further social inequalities among gender categories using an intersectional lens, such as age, marital status, education, income, or disability [8].

## Gender and feminist evaluation

Within public health, contemporary gender-sensitive evaluation can trace it roots back to international development and the Gender and Development (GAD) approach which emerged in the late 1990s [9,10]. This approach emerged out of a criticism of the Women in Development (WID) approach, which focused on the inclusion of women in development projects as a mechanism to address gender inequality. In addition to focusing on the exclusion of women in development, gender-sensitive evaluation is also interested in examining the structural inequalities and gender power relations between men and women as well as exploring the differential implications of development activities for men and women [10].

Feminist scholars have criticized gender-sensitive evaluation for not going beyond the consideration of inequitable gender power relations – feminist evaluation argues that inequitable gender power relations (and women’s position in society) also need to be challenged and changed [9,11–13]. Other important components of feminist evaluation include the engagement of women and communities within the evaluation process, the understanding that knowledge itself is a resource that belongs to the people who are targeted by the evaluation, and evaluators should be held accountable for change based on evaluation findings [9,13].

A number of scholars have attempted to reconcile and merge the two approaches. Kalpazidou Schmidt and Krogh Graversen [14] state that ‘gender-sensitive or feminist evaluation sees inequality as systemic and structural, and evaluation as a political activity’ and have proposed an associated conceptual evaluation framework for gender equality interventions. While Bustelo [11] proposes conducting evaluation from a gender+ perspective, which has ‘a structural and feminist understanding of gender inequality’. UN Women refers to a similar approach as gender-responsive evaluation [15]. They argue that gender-responsive evaluation ‘assesses the degree to which gender and power relationships – including structural and other causes that give rise to inequities, discrimination and unfair power relations, change as a result of an intervention’ [15]. For UN Women, how the evaluation is conducted is just as important as to what it examines, and a gender-responsive evaluation should be undertaken ‘using a process that is inclusive, participatory and respectful of all stakeholders’ [15].

The changes in how gender is conceptualized in regard to evaluation reflect how thinking around gender analysis has changed over the last two decades and the associated tools and frameworks. Within public health, for example, many argue that an intersectional approach to gender analysis is vital as it is no longer enough to explore how gender power relations manifest as inequities between men and women, but also among different groups of men and women [16]. In addition, we must seek to also understand how and where gender power relations are negotiated and changed. Morgan et al. [5] present a gender framework which not only looks at what constitutes gender power relations, but also where and how power is negotiated and changed (at the individual/people and structural/environment level). The Gender Integration Continuum framework developed by the USAID’s Interagency Gender Working Group (IGWG) [17] is a commonly used tool to integrate gender into public and global health programs. It conceptualizes gender integration on a continuum – from gender exploitative, to gender accommodative, to gender transformative. Its applicability to gender and evaluation lies in the fact that gender-sensitive evaluation is similar to gender accommodative approaches, while feminist evaluation is similar to gender transformative approaches. Understanding gender integration on a continuum allows for the possibility that a program or evaluation (or components of a program or evaluation) can lie in multiple places at once, or lie in the center of the two approaches. UNICEF’s approach to gender integration in evaluation supports this understanding [18]. They argue that applying ‘a gender lens to the evaluation process supports a proper analysis of how unobserved gender norms and gender discrimination can affect programme implementation processes and outcomes for diverse groups of women and girls, and men and boys’ with the aim of improving the quality of an evaluation and providing the ‘basis for more gender-transformative programming towards achieving gender equality goals’ [18]. The International Center for Research on Women (ICRW) takes a similar approach and provides relevant examples of how gender-responsive monitoring can be implemented in low- and middle-income country settings [19,20].

The gender integration steps described in this paper build off existing guidance on integrating gender into public health interventions, such as MEASURE Evaluation’s Seven Steps to EnGendering Public Health Evaluations [21]. Similar skills are discussed, such as selecting indicators that go beyond sex disaggregation to measure gender constructs. The guidance provided here, however, provides a step wise approach to systematically integrating gender into evaluations, offering practical tools to help with this process. We share UNICEF’s understanding of gender integration, which seeks to understand how gender power relations affect implementation processes and outcomes with the aim of improving an intervention or programme’s implementation process while providing the basis for more gender-transformative programming. We see gender-sensitive and feminist evaluation to be complementary and on a continuum as opposed to being separate approaches. Integrating gender into an evaluation allows us to identify how gender power relations might impact the ability of a programme to meet its objectives, and modify implementation accordingly. It also allows us to explore whether our programming may unintentionally exacerbate existing gender inequities. By doing so, we can not only improve the effectiveness of our interventions, but move towards building interventions which are transformative in nature.

## Integrating a gender lens into the RADAR maternal health coverage survey

The activities described in this paper were conducted to incorporate a gender lens into a RMNCAH coverage survey and facilitate gender analysis of data for the Real Accountability: Data Analysis for Results (RADAR) project [22]. RADAR is an initiative funded by and implemented by Johns Hopkins University, Institute for International Programs (JHU-IIP) to increase the availability of reliable data for low- and middle-income country (LMIC) programs in RMNCAH and to promote evidence-based decision-making. The RADAR project specifically focuses on improving the implementation of rigorous evaluation of RMNCAH programs. While the coverage survey has since been implemented and analyzed by country partners and the internal team [23], the guidance here is not discussed in relation to one particular intervention.

RADAR worked with RMNCAH partner organizations to develop a framework to guide program evaluation, accompanied by five core evaluation questions. The framework and questions are further discussed the paper by Amouzou et al [24] which is part of this series. Through the development of a suite of compatible evaluation tools, RADAR worked with partner organizations to collect, analyze, and use data related to these questions, along with associated indicators and methodologies, in order to improve RMNCAH programming. Tools included: a coverage survey, an evaluation planning tool, an implementation strength assessment survey, a quality of care survey, and the Lives Saved Tool (LiST), which estimates the impact of scaling up RMNCAH interventions. The tools are meant to be used to evaluate a variety of RMNCAH programs and were not designed for one program in particular.

**Box 1.** Five Core Evaluation Questions about RMNCAHN Programs.

Question 1: Does the program focus on interventions that will have the greatest impact in the program context? Question 2. Is the pathway to RMNCAH&N impact clearly developed and evidence-based? Question 3. Is the program being implemented as planned, and at sufficient strength and quality to achieve expected impact? Are services being utilized? Question 4. Do women and children who need interventions actually receive them? Question 5. Is the expected impact of the program occurring? Why or why not?

Integrating a gender lens into the RADAR tools involved the following overarching activities: (1) incorporating gender-sensitive indicators and questions relevant to RMNCAH into evaluation tools; (2) ensuring that results profiles are disaggregated by sex and other relevant social stratifiers whenever possible and that key gender equity issues are included within the reporting; and, (3) the development of a men’s survey to correspond with gender-related questions in the women’s questionnaire. To facilitate this work, a model was developed that explored how gender power relations manifest at the community, organizational and service delivery level, which map directly onto the coverage, implementation strength, and quality of care tools and outline how gender inequity may affect implementation at each level. Indicators were mapped against the associated gender model and, where relevant, questions were added to the tools.

The steps and tools discussed in this paper were used to incorporate a gender lens into a coverage survey for maternal health, however, they can also be used to incorporate a gender lens into any evaluation tool. Within RADAR, the coverage survey was meant to be utilized by multiple country partners implementing diverse maternal health programs, as opposed to being attached to one particular program. Coverage surveys are used to ensure that interventions reach their intended population and can be implemented prior to and/or after program implementation. They allow researchers and implementers to assess health services across a population and, by exploring factors associated with access to health interventions or services, examine why individuals may not have received an intervention or treatment. The findings can then be used to influence activities aimed at increasing the reach of interventions/services, particularly if current coverage is not meeting its intended target. It is important to note that the modifications made to the RADAR evaluation tools happened during the analytical stage of evaluation, and not during other stages, such as identifying evaluation users or during implementation. For evaluators looking to integrate a gender lens into their evaluations at different stages, other tools should be consulted [18,21].

## Steps to integrate a gender lens into the RMNCAH coverage survey

Four steps are presented below, which will help evaluators integrate gender into their RMNCAH tools: (1) Choosing the right variables for data disaggregation; (2) Using a gender framework and matrix to identify the ways in which gender inequities manifest as root causes of mortality or morbidity; (3) Identifying gender equality outcomes, gender indicators and gender analysis questions for inclusion with evaluation tools; and, (4) Engaging men through a men’s survey. Methodological considerations for implementing robust evaluations and surveys are discussed in Amouzou et al and Munos et al in this special issue and should be taken into account in order to ensure a population-based sample is achieved to minimize selection biases in participation of men and women in the survey.

### Step 1: choosing the right variables for data disaggregation

Ensuring data is disaggregated by sex and other biological and social stratifiers is an important step in gender analysis. It is important to note, however, that sex disaggregation is not in and of itself gender analysis, but an entry point for gender analysis. Sex disaggregated data is important as it can show where differences between men and women or boys and girls exist. When both men and women or boys and girls are included in a program, intervention data should always be disaggregated by sex in addition to other biological and social stratifiers, such as age, income, disability, race, ethnicity, migrant status, sexual orientation, geographic location, etc., to allow for the incorporation of an intersectional lens and explore within group differences. This is referred to as intersectional sex disaggregated data. In some instances, interventions and programs may only target women or men (referred to as sex specific data). In such instances, data should still be disaggregated by other relevant biological or social stratifiers. This is referred to as Intersectional sex specific data. Gender analysis can be incorporated into studies which use intersectional sex specific and intersectional sex disaggregated data. Which variables that are chosen will depend on the context in which the evaluation is taking place, in addition to considerations related to feasibility. For additional guidance on choosing the right variables and data disaggregation, additional guidance should be consulted [see: 18,19,23].

### Step 2: using a gender framework and matrix to identify the ways in which gender inequities manifest as root causes of mortality or morbidity

Gender inequality, like other forms of inequality, are underlying causes for poor health outcomes [25]. Interventions which are gender responsive, and in some cases seek to change inequitable gender norms, roles, and relations, therefore have the potential to have long lasting change by addressing underlying systems and structures. By actively addressing gender inequality through increasing women’s access to resources, redistributing labor/work within and outside the home, challenging harmful gender norms, and increasing women’s autonomy and decision-making power, for example, RMNCAH programs can have a positive effect on health outcomes [4,26,27]. In some cases, the interventions which have the greatest impact may not be health interventions but gender equality and gender-responsive interventions, more broadly.

However, the ways in which gender power relations affect health program outcomes are often indirect, meaning that the causal pathway in which they affect health is not always obvious, i.e. it is not always easy to link gender equality/ inequity to mortality or morbidity. As a result, efforts to address health inequities or outcomes are often seen as different or separate from efforts to promote gender equality – often meaning that health interventions focus solely on health outcomes and not gender equality outcomes (a gender equality outcome is one in which measures equality between men and women, such as equitable access to resources or decision-making power). In order to understand the indirect ways in which gender inequity/inequality can affect mortality and morbidity so as to incorporate them to their impact models or other tools, evaluators can use a gender framework and gender analysis matrix, such as those described below.

Gender frameworks are used to explore gender power relations by breaking down the ways in which gender power relations manifest to create different and/or inequitable experiences and outcomes. Common frameworks include the Morgan et al framework [5] and the Jhpiego framework [7]. These present the different ways in which gender power relations manifest as inequities which can affect health and other outcomes, including differential: access to resources; labour, roles, and practices; norms, values, and beliefs; and decision-making power and autonomy [5].

A gender analysis matrix is a way of organizing information for gender analysis [28]. Matrixes can be used to identify key gender-related considerations, including barriers and constraints, relevant for a health or health system area and/or to develop gender analysis questions and indicators for inclusion in a program’s implementation and evaluation tools (discussed in step three below). Using a gender analysis matrix will allow evaluators to ensure their evaluations are gender responsive in a systematic and comprehensive way. A gender analysis matrix can be used to: identify how gender analysis can be conducted within existing data sets, identify key gender-related considerations for evaluations, and/or how evaluations can be modified to take into account such considerations. As such, a matrix can be used to identify and/or develop: gender considerations (barriers and constraints which may affect outcomes), gender equality outcomes, gender analysis questions for inclusion in data collection tools, and gender indicators for monitoring and evaluation.

Table 1 presents an example of a gender analysis matrix. Gender analysis matrixes are meant to be modified to meet study needs and objectives; the topic domains should be modified to relate to areas of consideration within an evaluation and can be based on the aims and objectives of the evaluation or a recognized framework. The topic domains included in the matrix in Table 1 are: access to and utilization of services, quality of care – provider-patient interactions, and facility/ infrastructure. The gender analysis domains included within a gender analysis matrix should remain consistent with a recognized gender framework. The gender analysis domains included in Table 1 are: access to resources, distribution of labour, practices, roles, norms, values, beliefs, and decision-making and autonomy. The questions within each domain are meant to provide examples of the types of questions that can be asked and are not meant to be exhaustive. Answers to the questions in the sex/gender disaggregated data column include quanitifiable information on differences and inequalities between and among women and men. For example, these questions may explore whether there are differences in morbidity and mortality or in access to health services from baseline to endline of the program implementation between and among women and men. Answers to questions within the gender analysis domains columns can help explain differences seen within the sex/gender disaggregated data column. Due to the context specific nature of gender power relations, not all questions will be relevant for all contexts. Potential data sources are included next to each question.

**Table 1.** Example gender analysis matrix.

Topic Domains	Sex/ gender disaggregated data	Gender analysis domains			
		Access to Resources	Distribution of Labour, Practices, Roles	Norms, Values, Beliefs	Decision-making power, Autonomy
**Access to and utilization of services**	Percentage of men and women accessing/using services. How does this differ between different groups of men and women? *(data source: household survey)*	To what extent do men and women have access to knowledge about services? How does this differ between different groups of men and women? *(data source: household survey)*	Are there occupational or household activities that prevent men and women from accessing and using services? *(data source: household survey; qualitative inquiry)*	Do gender norms affect men’s or women’s willingness or ability to utilize services? How does this differ between different groups of men and women? *(data source: qualitative inquiry)*	Who decides whether or not someone can participate in screening – and at what level, i.e. within households, communities, institutions? *(data source: household survey; qualitative inquiry)*
**Quality of care – provider-patientinteractions**	Number of female providers available. *(data source: facility survey)*	To what extent do men and women have in-person contact with a health provider?	To what extent is respectful maternity care practiced? *(data source: facility survey; observational data)*	Are women who are accompanied by their male partners treated differently from those who are not? *(data source: facility survey; observational data)*	Do female community health workers have the autonomy to make decisions related to service provision? *(data source: qualitative inquiry)*
***Facility/ infrastructure***	Distance of health facilities. *(data source: facility survey)*	How do the conditions at health facilities affect access to and utilization of services? To what extent do health facilities provide services with appropriate conditions (such as functioning toilets, bathing areas for inpatient facilities, shelter from sun/rain in the waiting area) and confidential services?	Are there female health providers available? *(data source: facility survey)*	Can patients request to consult a health care provider of their choice if they prefer to? *(data source: facility survey; observational data)*	Is there a policy requiring male partners to accompany women? *(data source: document review; qualitative inquiry)* Do hours of operation affect men’s and women’s ability to access services? (data source: qualitative inquiry)

### Step 3: identifying gender equality outcomes, gender indicators and gender analysis questions for inclusion within evaluation tools

As discussed above, the ways in which gender power relations affect health program implementation and health outcomes are often indirect and multifaceted. Gender indicators and questions included within evaluation tools need to unpack the different ways in which gender power relations manifest as inequities to affect mortality and morbidity, such as through differential: access to resources; roles and practices; norms, values and beliefs; and decision-making power and autonomy [10,14,29]. As such, proxies for gender equality outcomes are used to explore the different ways in which gender power relations manifest. For example, differential access to resources can be explored through questions related to access to income, education, or technology between and among men and women. In coverage surveys, to explore whether access to and utilization of a particular resource has an impact on a health outcome, for example, you would assess whether there is an association between a gender equality outcome (e.g. access to income) and the health outcome (e.g. maternal mortality). Due to the multi-faceted nature of gender power relations, when conducting a gender analysis, it is important to look across multiple gender domains (e.g. access to resources, roles and behaviors, norms and beliefs, and decision-making power) and the ways in which they may interact.

Table 2 presents examples of gender equality outcomes related to each gender analysis domain which were used in the coverage survey. These can then be converted to gender indicators for inclusion within evaluation surveys.

**Table 2.** Gender equality outcomes.

Access to Resources	Roles and Practices	Norms & Beliefs	Decision-making and Autonomy
Woman has own money Woman worked last week Woman worked last year Woman has access to mobile banking Woman has own bank account Woman has a mobile phone	Woman’s husband/partner attended ANC Woman’s husband/partner attended delivery Husband attended health facility for family (child or wife)	**Positive outcomes** The husband should accompany to ANC The husband should accompany to delivery Contraception is women’s concern Woman has the right to refuse sex with her husband **Negative outcomes** Husband is justified in beating wife for any reason Childbearing is women’s concern If a woman refuses sex, her husband has the right to: reprimand/get angry with her; refuse money; use force for sex; have sex with another woman	Woman is able to leave the house for any reason Woman can make own decisions about health Woman can make major purchase decisions Woman can make decisions to visit family or relatives Who should sell poultry (women/both) Who should sell livestock (women/both) Who should decide to visit wife’s family (women/both) Who should decide how many children (women/both) Women can decide how to use her own money

A gender analysis matrix (discussed above) can help evaluators identify gender equality outcomes. Once appropriate gender equality outcomes have been identified, these can then be converted into gender-responsive indicators and gender analysis questions for inclusion within evaluation tools. Gender-responsive indicators include sex-specific and/or sex-disaggregated indicators, as well as gender equality indicators which explore the role of gender inequality in relation to particular health or health system outcomes [29]. Sex-specific indicators pertain to only women or only men and seek to explore differences among different groups of women or men; sex-disaggregated indicators measure differences between and among women and men in relation to a particular metric; and gender equality indicators measure gender (in)equality directly or as a proxy for gender (in)equality. Note that, similar to sex-specific and sex-disaggregated data, including only sex-specific or sex-disaggregated indicators does not constitute a full gender analysis but rather is an entry point for it. When using sex-disaggregated or sex-specific indicators be sure to disaggregate data further by other relevant biological or social stratifiers. In addition, it is important to note that one gender-responsive indicator may need multiple questions within an evaluation tool to be able to address it. Table 3 provides examples of gender indicators and gender analysis questions in relation to some of the gender equality outcomes outlined above.

**Table 3.** Gender equality outcomes, indicators, and questions.

	Gender Domains			
	Access to Resources	Roles and Practices	Norms & Beliefs	Decision-making and Autonomy
**Gender Equality Outcomes**	Woman has own money	Woman’s husband/partner attended ANC	Husband is justified in beating wife for any reason	Woman is able to leave the house for any reason
**Indicator(s)**	Percentage of women age 15–49 who have done any work in the last 7 days Percentage of women age 15–49 who have done any work at any time in the past 12 months Percentage of women age 15–49 who earned cash for work at any time in the past 12 months	Percentage of women who were accompanied by their husbands/partners to an antenatal care (ANC) visit Percentage of women whose husbands/partners were present during the antenatal care (ANC) visit consultation	Percentage of women age 15–49 who think a husband is justified in hitting or beating his wife under certain circumstances	Percentage of women age 15–49 who are usually permitted to go to specific places outside of their home alone
**Question(s)**	Aside from your own housework, have you done any work in the last seven days? As you know, some women take up jobs for which they are paid in cash or kind. Others sell things, have a small business or work on the family farm or in the family business. In the last seven days, have you done any of these things or any other work? Have you done any work in the last 12 months? Are you paid in cash or kind for this work or are you not paid at all?	Did your husband/partner accompany you in any antenatal care visits during this pregnancy? Was your husband/partner present in the room or other space during your antenatal care consultation?	In your opinion, is a husband/partner justified in hitting or beating his wife in the following situations: If she goes out without telling him? If she neglects the children? If she argues with him? If she refuses to have sex with him? If she burns the food? If she refuses to give her earned money to her husband/partner? If she uses contraception without informing her husband/partner?	Are you usually permitted to go to the following places on your own, only if someone accompanies you, or not at all? To the local market to buy things? To a local health center or doctor? To the community center or other nearby meeting place? To homes of friends in the neighborhood? To a nearby shrine/mosque/temple/church? Just outside your house or compound?

### Step 4: engaging men through a men’s survey

Due to the nature of gender power relations, undertaking a men’s survey may also be an important tool for unpacking power dynamics and relationships between gender equality outcomes. A men’s survey was incorporated into the RADAR Coverage Survey and included similar questions to the women’s survey [30]. Men’s and women’s surveys can be analyzed separately or as pairs, i.e. in aggregate regardless of household, or within the same households or relationship/marriage. Unpaired analyses between men and women in an area may reflect overall average trends between gender norms and perceptions among men and women’s health outcomes in a given community. While paired analysis can help examine potential pathways that may benefit from an intervention for health outcomes. Pairing responses may limit the sample size for some analyses: for example, understanding how men’s perceptions of gender norms and the effect those have on RMNCAH outcomes provides a more robust and complete understanding of how gender inequality affects health outcomes.

## Conclusion

Incorporating gender into RMNCAH evaluation tools is important in order to understand how gender power relations may affect program implementation and all programs can benefit from a gender lens regardless of whether they are specifically targeted at understanding the role of gender power relations [18]. Incorporating gender power relations into evaluation tools, however, requires careful planning and integration from the initial steps in RMNCAH program development and throughout all stages of program implementation. Incorporating gender equality outcomes, indicators, and questions into program evaluation tools, in addition to disaggregating data by sex and other biological and social stratifiers, and analyzing data through a gender lens, can enable evaluators to understand the ways in which gender power relations are affecting program implementation and outcomes. By identifying gender barriers that affect access to and utilization of programs, for example, evaluators can modify interventions accordingly while also ensuring that their programs are not unintentionally exacerbating existing gender inequities, which ultimately lead to more effective and high impact interventions to improve population health and well-being.

## Paper context

Guidance on how to integrate a gender lens into research, interventions, and policy is often vague and gender analysis is applied in an unsystematic way. The paper provides researchers with guidance on how to integrate gender into their evaluation tools by outlining a four-step process which allows researchers to comprehensively and systematically integrate gender analysis into their evaluations.
